# Trends in Determinants of Entry into the Academic Career: The Case of South Korea, 1980-2010

**DOI:** 10.1371/journal.pone.0141428

**Published:** 2015-10-28

**Authors:** Keuntae Kim, Jong-Kil Kim

**Affiliations:** 1 Department of Sociology, University of Wisconsin-Madison, 2445 William Sewell Social Sciences Building, 1180 Observatory Drive, Madison, Wisconsin, 53706, United States of America; 2 Department of Sociology, Duksung Women’s University, 33 Samyangro 144-gil, Dobong-gu, Seoul, Korea; Central South University, CHINA

## Abstract

Substantial research documents the determinants of entry into the academic career, yet little is known about how these determinants have evolved over time. Using data from a large sample of Korean scholars who received their doctoral degrees between 1980 and 2010, we estimate discrete-time event history models of transitioning to an academic position in any academic field. Results indicate that universalistic characteristics, such as publication record, strongly affect subsequent career success, but so do particularistic factors, including doctoral institution prestige. Since the 1980s, the influence of doctoral degree prestige increased substantially more than the influence of one’s publication record on higher education employment, implying that the rising importance of particularistic factors has outpaced growing consideration of universalistic characteristics in Korean academia. However, the importance of gender on academic employment has declined since the early 2000s, suggesting that the implementation of employment quotas for female professors may have stymied gender discrimination.

## Introduction

Higher education institutions play a crucial role in determining a nation’s international competitiveness. Universities not only provide facilities for scholars and researchers, but they also encourage the advancement and development of knowledge and its application to government, industry, and community. As professors are central to these processes, recruiting new faculty with strong academic potential is essential for universities to fulfill their primary social functions. For these reasons, Merton argued that the modern university system should hire professors on the basis of merit, a principle which he calls *universalism* [1]. He maintained that the evaluation of academic achievements and the allocation of academic rewards, including employment decisions, should be based on universalistic criteria that capture generalized academic competence rather than functionally irrelevant characteristics, such as sex and race. Despite Merton’s assertion that universalism constitutes a distinctive ethos of the scientific community, however, a number of empirical studies have found that doctoral candidates’ job placement is influenced more strongly by status attributes, such as their academic advisor’s reputation, than by their academic achievements, for example as measured by publication record [2], [3], [4], [5], [6], [7].

Since the end of the Korean War, the South Korean higher education system has been closely modeled after the U.S. system and has expanded tremendously over the last several decades [8], [9]. Discourse on academic appointments in Korea emphasizes universalism as an ideal, but there is evidence that particularistic factors, such as the prestige of a scholar’s undergraduate university or alumni networks, influence job placement in the South Korean academic labor market [10]. Nonetheless, few studies have empirically assessed the influence of universalistic versus particularistic factors in attaining an academic appointment in Korea.

Furthermore, the results of past research are not widely generalizable across time or academic discipline. For the most part, prior research relies on cross-sectional data, examining correlations between individual scholars’ attributes and employment outcomes at a single point in time. Consequently, these studies do not address the possibility that primary determinants of employment may evolve over time. Such an omission is unfortunate given the changing normative climate surrounding academia, which may have accelerated in recent decades [11]. In addition, past studies largely focus on small cohorts of scholars in the natural sciences [12], [13], [14], [15], while relatively little is known about academic job markets in other fields, such as the social sciences and humanities, which may have different systems for allocating rewards.

To address these gaps in our knowledge, the present analysis draws on data from a large sample of scholars across all academic fields in Korea and who received their doctoral degrees between 1980 and 2010. During this period, not only did Korean institutions of higher education grow rapidly, but various policy measures were enacted to ensure equal opportunities in academic employment. These include a faculty recruitment quota system limiting the number of people that may be hired from certain universities [16], as well as gender quotas that limited the proportion of men that may be hired in national universities [17]. For these reasons, the 1980–2010 Korean academic job market is an ideal case to explore the evolution of hiring academic hiring practices—specifically, the reliance on universalistic versus particularistic characteristics for determining academic appointments—in a rapidly changing environment.

## Literature Review

### Higher Education in Korea

**Fig 1:**
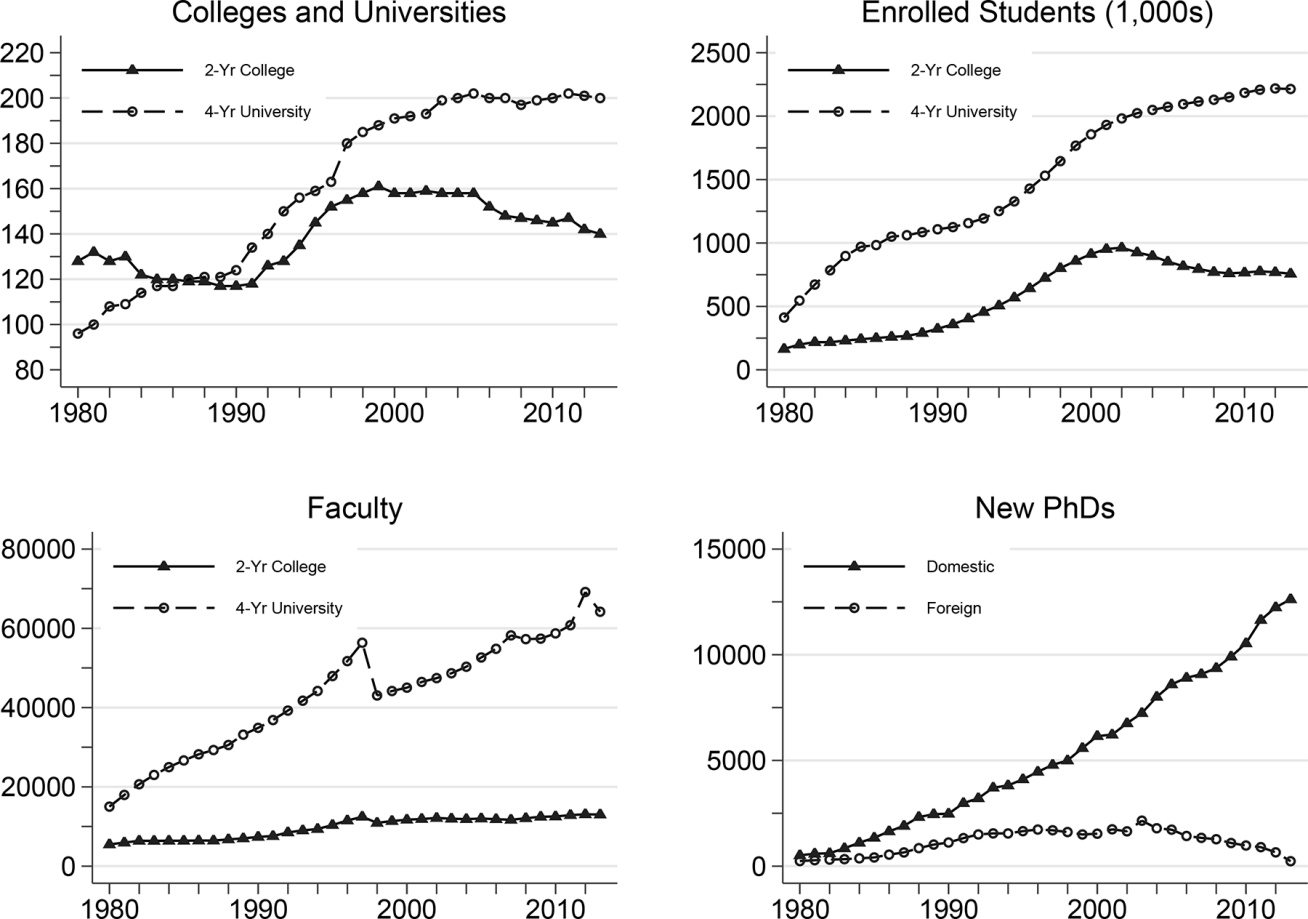
Trends in Higher Education in Korea, 1980–2010. Statistical Yearbook of Education 1980–2013, Department of Education, Seoul, Korea.

Over the past seven decades, Korea has experienced a dramatic expansion of higher education at a rate unmatched by other Asian countries [18]. As illustrated in Fig 1, Korea contained 128 two-year colleges and 96 four-year universities in 1980. By 2013, while the number of two-year colleges had increased slightly to 140, the number of four-year universities more than doubled to 200, with particularly fast growth during the late 1990s when the establishment of new institutions was deregulated. The number of Korean students enrolled in higher education was approximately 0.6 million in 1980, compared to roughly 2.9 million in 2013. The increase in student enrollment was especially pronounced during the early 1980s when the Korean government increased university admission quotas, though still requiring universities to drop a fraction of students before graduation. However, the graduation quota policy was aborted after a few years due to enormous opposition from professors and administrators [18].

Along with this growth in universities and enrolled students over the past three decades, the number of full-time faculty has tripled, from 20,510 in 1980 to 77,208 in 2013. The total number of academic employees at higher education institutions (including part-time lecturers and non-tenure-track professors) has grown by 3.5 percent per year since 1990 [19]. Furthermore, the number of doctoral degrees conferred by domestic institutions has increased more than twenty-fold since 1981, from 589 to 12,625 in 2013 [20]. During the same period, the number of Koreans earning doctoral degrees abroad reached a peak at 2,152 in 2003, and the number has since declined.

In the context of massive growth over a relatively short time period, processes of job attainment and promotion in the Korean higher education system have been shaped by unique features of its historical development. These include its privileging of prestigious undergraduate and graduate institutions, in particular degrees conferred by U.S. institutions, as well as its emphasis on seniority. Although research on the determinants of academic appointments in Korea is quite limited, available results mostly align with those for Western countries, where both institutional prestige and seniority are key factors shaping job outcomes in academia [21]. For example, research shows that, as in Western countries such as the U.S., undergraduate university prestige has a substantial impact on the likelihood of academic appointment in Korea [22]. Baccalaureate degrees from prestigious universities can even offset a weaker scholarly publication record due to the prevailing perception that job candidates from prestigious universities will ultimately produce more upon appointment than those from less prestigious universities.

Despite consistency in the key determinants of career advancement in Korean and Western higher education institutions, these processes play out in unique ways in the context of the Korean academic system. The most prestigious universities in Korea ‒ Seoul National University, Korea University, and Yonsei University ‒ are commonly referred to as “SKY” universities, not only as an acronym but metaphorically, because they are hard to reach like the sky. In addition to SKY universities, Korea has two new elite universities, Korea Advanced Institute of Science and Technology (KAIST) and Pohang University of Science and Technology (POSTECH), which are benchmarked against the Massachusetts Institute of Technology (MIT) and the California Institute of Technology (Cal-tech). Graduate programs were first established in these elite universities after the Korean War, while non-elite Korean universities launched their doctoral programs mostly in the 1970s and 1980s. As a result, most faculty members in Korean universities first established during this time period were scholars who graduated from the SKY universities and, through their strong alumni networks, these professors continued to recruit scholars from their alma maters. This strong preference for peer alumni drove the emergence of a Korean “academic caste system” [4], in which one’s undergraduate institution asserts a considerably stronger influence on academic appointment than in Western countries.

One of the most consistent findings in past research is that doctoral degrees earned from overseas institutions, particularly in the U.S., have a decisive impact on academic employment in Korea [23], [22]. As in other Asian nations, in Korea all aspects of contemporary academic institutions–such as systems of institutional governance, the ethos of the academic profession, and the curriculum–have roots in Western academic models, particularly the American model [8], [9].

Following the end of the Korean War, the U.S. Military Government sent Korean students to the U.S. in hopes that they would return to Korea and contribute to rebuilding the national education system. As intended, many of these U.S.-trained scholars returned to Korea and were hired as faculty in higher education institutions, where they played leadership roles in reshaping the structure of university administration, governance, financing, and curriculum development [9]. Since then, the brightest Korean students, who eventually become leading professors in their home country, have continued to enter doctoral programs in the U.S. due to Korea’s relatively less developed graduate education system and better funding prospects and social networking opportunities abroad. Consequently, in 2013 about 19,916 Korean students were enrolled in graduate programs at U.S. higher education institutions. Since 2002, among foreign countries, Korea has had the third highest number of students studying at the tertiary level in the U.S., trailing behind only China and India [24]. Accounting for the substantially larger populations of China and India further highlights the massive proportion of Korean students entering U.S. graduate programs. It is argued that Korea’s reliance on the U.S. for graduate training is so pervasive that it can be characterized as intellectual colonialism, in that the intellectual foundation of science is located in developed countries (primarily the U.S.) without fundamental reflections on the knowledge production system [23].

Under these circumstances in Korean, it is not surprising that U.S.-educated faculty overwhelmingly outnumber those who earned doctorates elsewhere, including from domestic institutions. In 2008, 72.8 percent of all professors of public administration at the 25 major Korean universities obtained their doctorates in the U.S. [23]. Moreover, these U.S.-trained scholars, who often play leadership roles in Korean academia, maintain their high social status by excluding those who earned doctorates elsewhere [22]. Recognizing the significant disadvantages attached to doctoral degrees earned in domestic institutions, brighter students choose the U.S. for their doctoral study. This has transformed the graduate student body in Korea into one consisting mostly of individuals still working for pay outside academia [25], [23].

In addition to perceptions of institutional prestige, seniority privileges are another source of disadvantage for Ph.D.s from domestic institutions (and, to a lesser degree, from foreign institutions other than U.S. institutions). Seniority is a core principle of organizational norms in Korean society more broadly, playing an important role in both public and private sector organizations. Following suit, higher education institutions in Korea have long maintained wage and promotion systems based on seniority. Although seniority is also a critical factor determining wages and promotions in the U.S. academic system, it is much more salient in Korea [26], [27]. In Korea, job candidate age is a key consideration in academic appointment decisions, where the likelihood of a job offer is considerably reduced for applicants who are older than the youngest faculty member in the department. Approximately 80 percent of students enrolled in Korean graduate schools in 2008 were enrolled part-time [25]. Because part-time enrollment tends to extend time-to-degree, Ph.D. graduates from domestic institutions tend to be older than their foreign-Ph.D. counterparts when seeking their first faculty appointments [19]. This is one reason that some Korean faculty recommend that their advisees go on to doctoral programs in the U.S. after completing a master’s degree in Korea.

### Determinants of Entry into an Academic Career

A substantial body of research has assessed processes of stratification in science through the universalism-versus-particularism model [28]. According to the universalism perspective, the scientific community should recognize and reward individuals on the basis of the quality of their contributions to scientific knowledge, and not on the basis of their personal attributes, such as age, gender, class, or race/ethnicity [1]. In universalistic systems, objective accomplishments serve as the basis for marking one’s worth. In contrast, in a particularistic system, evaluation and decision making is based on anything *other than* universalistic factors.

One of the most crucial forms of scientific contributions that hiring institutions may reward is publication record, widely considered the most direct reflection of a researcher’s competence as a scientist [12]. Despite the theoretical expectation that publication record positively impacts the likelihood of academic appointment, empirical evidence is mixed. On the one hand, a number of studies find no or little effect of pre-employment research productivity on the probability of academic appointment after accounting for particularistic factors, most notably the prestige of the doctoral department [29], [5], [13]. On the other hand, some scholars [30], [31] argue that universalistic influences, in particular the number of publications as a Ph.D. student, have a much stronger impact than particularistic factors, although the best fitting model included both universalistic and particularistic factors.

Another universalistic factor considered in the process of obtaining academic employment is years elapsed between the baccalaureate and doctorate, as well as age at receipt of doctorate, because these can be used as proxies for academic performance in graduate school [3]. The use of these factors as proxies is based on the assumption that talented students can complete degree requirements more quickly than their less talented peers. Several studies indicate that time spent in graduate school, as well as age at receipt of doctoral degree, are negatively associated with the prestige of the first academic position [3], [32], [33].

One of the most consistent findings in the literature on stratification in science is that the reputation of the doctoral department is the most powerful element predicting the prestige of the first academic job [3], [4], [29], [34], [13]. Net of the effects of the graduate institution, the prestige or selectivity of a scholar’s undergraduate institution is also significantly associated with academic employment, albeit to a lesser extent [32], [13]. Furthermore, once employed at a prestigious department, scholars gain access to more resources and rewards than those employed at less prestigious institutions, which, in turn, leads to more career success, including publications, academic recognition, and research grants [35], [36], [37]. Burris argues that, at the department level, social networks accumulated through interdepartmental hiring of Ph.D. students among elite departments and processes of social closure maintain the prestige hierarchy to such an extent that it resembles a caste system [4].

Gender is also one of the most important factors in particularistic models. A large body of research has found that, on average, women achieve significantly lower levels of career success than their male counterparts, even after accounting for publication record. The effect of gender on career outcomes is relatively small when factors such as marriage and childbearing are taken into account, and its effect diminishes substantially after initial hire; however, considered cumulatively, gender significantly shapes scientists’ careers [31], [21], [38].

It should be noted, however, that assumptions about the degree to which particular characteristics capture universalistic versus particularistic factors may be off base. For example, it is possible that doctoral department prestige reflects scholarly competence, if more prestigious graduate departments in fact tend to produce the most qualified job candidates. If this is the case, then doctoral institution prestige would reflect both universalistic and particularistic factors [39]. On the other hand, a great deal of research finds little direct association between doctoral department prestige and measures of scholarly productivity, such as number of publications, number of citations, or years elapsed between doctorate and employment [3], [40], [14].

### Changes over Time

Although these universalistic and particularistic characteristics are fairly well established as key determinants of academic employment, few studies have examined trends over time in their relative impacts. Understanding how the relative influence of universalistic versus particularistic factors has evolved over time informs our understanding of the reward structure in science and illuminates processes of social stratification in academia. There are two hypotheses about how the relative reliance of labor market on universalistic vs. particularistic factors will have changed over time. One perspective predicts that labor market will increasingly rely on universalistic rather than particularistic factors, under observed changes in supply and demand, changing levels of uncertainty in labor markets, and/or industrialization and development. The other perspective predicts that reliance on particularistic factors will persist until the labor market expands to a point where the demand for jobs among the advantaged class reaches saturation.

According to a simple labor market model, when demand for faculty outweighs supply, new Ph.D.s are more likely to receive job offers than when demand falls short of supply [41]. Prior literature also suggests that, in periods when the academic job market is shrinking, the standards for faculty selection, promotion, and tenure become more demanding and rigorous [42], [43]. For instance, in the case of sociology, Bauldry reported that the median number of scholarly publications among newly hired assistant professors has doubled or tripled (depending on the measure of productivity) between 2007 and 2013, largely due to tightening of the job market since the U.S. financial crisis of 2008 [44].

Furthermore, Konrad and Pfeffer argue that the strength of association between productivity and rewards will decrease under conditions of uncertainty and ambiguity of job attainment and promotion [26]. Consequently, they suggest, in environments such as the contemporary academic market, increased competition will result in decreasing reliance on particularistic characteristics for allocating rewards and other resources because such practices become too costly. Long and Fox add that particularistic factors increasingly influence academic recruitment decisions when information about job candidate’s skills and abilities is more limited, the scientific paradigm is less developed, and secrecy is heightened [28].

Taken together, these theories imply that formalized recruitment practices with clear standards for evaluation, adequate information, and monitored decision-making processes encourage more universalistic patterns of hiring [2]. This prediction is analogous to modernization theory, which suggested that industrialization and the development of modern society would cause occupational achievements to increasingly result from *achieved* rather than *ascribed* characteristics [45]. Indeed, in Korea, some evidence suggests that academic hiring processes have become more transparent and evaluation standards more regulated since the late 1990s [19]. According to this line of reasoning, under these changing market conditions in Korean academia, academic employment should have become less dependent on particularistic factors and more dependent on universalistic factors over this time period.

Despite these theoretical expectations, numerous studies find that socioeconomic inequalities by ascribed attributes have persisted or even worsened over time in various social arenas, including labor markets, despite efforts to establish egalitarian systems [46]. To explain these anomalies, sociologists have proposed several theories. Among them, the Maximally Maintained Inequality (MMI) hypothesis [47], originally devised to explain cross-cohort changes in the effect of social class background on educational attainment, reveals insight into the observed pattern across cohorts in the influence of universalistic and particularistic factors on the likelihood of attaining an academic position. In principle, the MMI hypothesis maintains that educational expansion is unlikely to reduce inequalities because the socioeconomically advantaged are better positioned than their less advantaged counterparts to take up the new educational opportunities that expansion offers; thus, the needs of the advantaged class will be met before those of the less advantaged [48], [49]. More specifically, the MMI theory predicts that, even during periods of expansion, inequalities will be “maximally” maintained until the participation rate for the most advantaged group reaches saturation. That is, inequality will diminish under expansion only when the demand for all members of the advantaged group have been met, at which point members of less advantaged groups can benefit from additional supply created through continued expansion.

We argue that the MMI hypothesis may shed light on processes of stratification within the institution of science. For our purposes, attaining a faculty position is the outcome of interest, rather than attaining secondary education, and the basis of inequality is not only socioeconomic background but any particularistic factors considered in faculty hiring decisions. MMI would predict that the substantial expansion of higher education in Korea, resulting in the expansion of job attainment opportunities, would not reduce inequalities by particularistic factors unless saturation was reached. That is, the importance of particularistic factors may remain the same or even increase until higher education expansion creates more job opportunities than Ph.D.s with advantageous particularistic characteristics—for example, all those from prestigious departments.

To summarize, according to existing theories, it is reasonable to expect that the relative reliance on universalistic versus particularistic characteristics in Korean academia may have increased, decreased, or stayed the same over the past three to four decades. Although theoretical predictions have been offered on both sides based on evidence from the U.S., they have not been empirically tested in Korea, where the academic market is considerably smaller, interdependence among faculty members is greater, and key decisions tend to be made by senior faculty with less input from junior professors. Hence, it is necessary to investigate which theoretical predictions are supported as the enterprise of science in Korea has evolved.

## Data and Measurement

### Data

This study draws on data from the Korea Researcher Information (KRI) database maintained by the National Research Foundation of Korea (NRF). Benchmarked to the National Science Foundation in the U.S., the NRF was established in 1977 to support research and development activities across all academic fields in Korea. In the 2012–13 fiscal year, the NRF funded approximately 3.2 billion dollars for research and education in science and engineering, and research communities in Korea rely heavily on funding from the NRF.

In addition to detailed information on publications and patents, the KRI database includes basic demographic information, such as sex and date of birth. It also provides detailed information about scholars’ postsecondary education at the baccalaureate, master’s, and doctoral levels, including the date that each degree was granted, institution name and country, and field of specialization (i.e., major).

After casewise deletion, the analytic sample consists of 57,227 researchers who received a Ph.D. between 1980 and 2010 and who were registered in the KRI database by March 2014 (all data underlying our findings are available in S2 File). Since the NRF requires that all recipients of research grants, including collaborators, be registered in the KRI database, it also includes some scholars of foreign nationality. Because this study examines the academic marketplace in Korea, individuals currently employed at foreign institutions are excluded from the analyses. Hence, Korean scholars employed at foreign institutions are excluded because their exposure to the academic job market in Korea may be limited. It should be noted, however, that registering with the KRI is not mandatory for individual scholars who are not recipients of research grants. Nonetheless, many researchers, including those working at private research institutes and graduate students, are strongly encouraged to register at the KRI when they work with faculty members in academic institutions.

### Measures

#### Prestige of Doctorate-Granting and Undergraduate Institutions

To measure the prestige of doctoral institutions, we first distinguished domestic institutions from foreign institutions. We then grouped domestic institutions into two categories: SKY universities, including KAIST and POSTECH, and all other universities in Korea. Foreign institutions are also divided into two categories ‒ one is the top 100 universities and the other is the non-top 100 universities ‒ using the *Academic Ranking of World Universities* (ARWU), which is updated annually by the Center for World-Class Universities at the Shanghai Jiao Tong University. ARWU rankings are determined based on university performance indicators such as the number of Nobel Prize winners, the number of articles published in top journals such as *Nature* and *Science*, the number of articles published in SCI and SSCI journals, and the average number of publications per faculty member. To minimize period effects, we measured university ranking as its average ARWU ranking over 2003 to 2013, the years for which the ARWU is available. We then selected the top 100 academic institutions using the ten-year-average annual ranking.

Combining the rankings for domestic and foreign institutions, we created an ordered categorical indicator of the prestige of doctorate-granting institutions, coded as follows: “1” for non-SKY domestic universities (the reference category), “2” for SKY domestic universities, “3” for world non-top 100 universities, and “4” for world top 100 universities. Hence, greater numbers correspond to higher prestige of the doctoral institution.

We measured prestige of undergraduate institution using the 2010 *Joong Ang Ilbo University Rankings*, which was modeled after the *U*.*S*. *News and World Report*’s university rankings. We created an ordered categorical measure with four categories: “1” for universities ranked 31 or lower, “2” for universities ranked 11–30, “3” for those ranked 6–10, and “4” for top 5 universities (i.e., the three SKY universities, KAIST, and POSTECH).

#### Number of Articles Published before Receipt of Doctorate

To assess academic performance prior to employment, we used individual-level data on the number of publications before receipt of the doctoral degree. Following the approach used in past studies [13], [21], we enumerated articles published only in journals listed in the SCI (Science Citation Index), SSCI (Social Science Citation Index), and KCI (Korean Citation Index). We considered articles published in the same year that the individual obtained his or her doctorate as pre-doctoral publications, under the assumption that these likely were accepted for publication pre-doctorate and thus may have influenced hiring decisions during initial job search. We excluded from our measure published books, book chapters, book reviews, non-academic reports, or essays because we had no way of ascertaining whether these publications underwent rigorous peer review, which is key to our assumption that publications indicate universalistic rather than particularistic factors. Despite evidence that authorship order influences academic employment (e.g., [13]), we did not restrict publications to first-authored articles due to our focus on initial employment. The vast majority of pre-doctoral publications are junior-authored works, often with students’ advisors or other doctoral committee members, likely because doctoral candidates have had less time to convert their research into publications than more experienced researchers. Thus, hiring committees are likely to consider co-authored and junior-authored publications when assessing the employability of recent Ph.D.s.

#### Other Variables

We derived a measure of the number of years elapsed between the baccalaureate and doctorate degrees by subtracting the year that the bachelor’s degree was granted from the year that the doctoral degree was obtained. Thus, this measure is only an indicator of time–to-doctoral degree because it does not account for when they started their graduate education, thus inflating time-to-degree for individuals who did not go to graduate school immediately after obtaining their bachelor’s degree. We excluded from our analysis individuals who took more than 40 years to complete their doctorate degree after their bachelor’s degree because they likely represent a different population of Ph.D.s, who may have substantially different characteristics than the average doctoral student.

We constructed a measure of age at receipt of doctorate by subtracting an individual’s year of birth from the year that the doctorate was received. We excluded from our analysis those who earned doctoral degrees before age 25 because they may represent a population with exceptional academic talents, or these may reflect data errors, both of which could produce biased results.

To measure gender, we relied on self-reports from the KRI, and we constructed a dummy variable coded “1” for females and “0” for males.

### Analyses

We employed logistic regression to estimate discrete-time hazard models of entry into first attainment of tenure-track academic position. We define tenure-track academic positions as holding an academic appointment at either a two-year college or four-year university. Although there might be some differences in hiring procedures between colleges and universities, professors employed at colleges and universities are not significantly different in terms of their educational qualifications or wages in Korea [19]. The discrete-time event history technique produces results almost identical to those of continuous-time hazard models [50]. However, the discrete-time event history approach is more straightforward than continuous-time models when incorporating time-varying covariates and age dependence into the model. The dependent variable in the discrete-time model is a dichotomous indicator of whether the respondent obtained a tenure-track job in an interval of two consecutive years, conditional on never having attained a tenure-track job at the beginning of the interval. Once employment occurs, individuals are no longer exposed to the risk of employment and are removed from the risk set. Additionally, individuals who did not experience employment by March 2014 (the date when data were extracted from the KRI) and those who were employed in non-tenure track positions were treated as censored at that year.

The effect of time, which is measured in years since doctorate receipt, is included in the model to capture the time dependence of the estimated hazard of first academic employment. The model includes both linear and quadratic terms to allow the hazard to change over time. Secular trends in the risk of employment over the periods are captured by including dummy variables for year of observation. Because time since the doctorate and the year of observation are not linear functions of one another, we can estimate the effects of both. Time and year of observation are time-varying covariates in the model. Furthermore, to account for discipline-specific unobserved traits that may affect faculty hiring processes, each model includes dummy indicators of academic discipline.

Instead of including interaction terms between independent variables and doctoral cohort, we estimate one model for each cohort with no period interaction effects. We report the coefficient estimates for each doctoral cohort because the primary purpose of the present study is to assess the extent to which each predictor’s influence changed over time, rather than testing only whether each is associated with the odds of employment. If the size of a coefficient for a variable changes over the periods, we would interpret this as evidence of a change in that factor’s impact over time.

## Results

Descriptive statistics for all variables in the analysis are presented in Table 1, delineated by doctoral cohort. Male Ph.D.s outnumber female Ph.D.s, and the proportion of female doctorate holders has increased more than twofold, from 13 percent in the 1980s to 30 percent in the 2000s. Age at doctoral receipt increased by approximately half a year in each decade, reflecting the aging structure of the academic profession [20]. In addition, years between the baccalaureate and doctorate increased during the periods under consideration; it now takes, on average, ten and a half years after graduating from a university to complete a doctorate. However, it appears that the prolonged age between the baccalaureate and the doctorate reflects the fact that students enter graduate schools at older ages than in the past, rather than that the number of years spent in graduate school has increased. Song and Kim found that duration in graduate school remained relatively stable while age at graduate school entrance increased over the periods [20].

**Table 1:** Descriptive Statistics by Doctoral Cohorts for Variables Used in Analysis of Entry into the Academic Career in Korea. Groups of related programs are presented here due to space limitations. In the analyses, 115 dummy variables for single instructional programs are used.

	1980–89		1990–99		2000–09	
	Mean	S.D.	Mean	S.D.	Mean	S.D.
Female	0.13		0.18		0.30	
Age at Receipt of Doctorate	33.50	3.95	34.17	3.96	34.87	4.69
Years between Baccalaureate and Doctorate	9.68	3.68	10.27	3.62	10.54	4.46
Number of Publications as Doctoral Student						
0 Publications	0.83		0.69		0.38	
1 Publication	0.07		0.12		0.14	
2 Publications	0.04		0.07		0.12	
3+ Publications	0.06		0.12		0.37	
Prestige of Undergraduate Institution						
Below Top 30 University	0.10		0.17		0.30	
Top 30 University	0.19		0.26		0.25	
Top 10 University	0.15		0.15		0.13	
Top 5 University	0.57		0.43		0.32	
Prestige of Graduate Institution						
Domestic, Non-SKY University	0.19		0.31		0.45	
Domestic, SKY University	0.22		0.26		0.28	
Abroad, Non-World Top 100 University	0.30		0.24		0.16	
Abroad, World Top 100 University	0.29		0.19		0.12	
Academic Disciplines						
Engineering	0.26		0.25		0.24	
Education	0.03		0.05		0.05	
Social Sciences	0.17		0.18		0.16	
Arts and Physical Education	0.01		0.02		0.04	
Medical Sciences and Pharmacy	0.12		0.09		0.11	
Humanities	0.11		0.18		0.16	
Natural Sciences	0.30		0.22		0.23	
*N*	6,577		22,712		27,938	

Descriptive results also indicate that doctoral students’ publication records improved dramatically over the periods under consideration. Among those who earned their doctorate during the 1980s, only 17 percent published at least one article at SCI (SSCI) or KCI journals, whereas 62 percent of those who obtained doctoral degrees during the 2000s did so. This may reflect a tightening of the academic job market, as hiring institutions now require higher publication standards.

As the number of doctoral degree-granting institutions increased, diversity in undergraduate institutions also increased. Students who graduated from below top-30 universities comprised only 10 percent of all Ph.D.s graduating during the 1980s; in the 2000s, the proportion for this group increased to 30 percent. At the same time, 57 percent of Ph.D.s during the 1980s graduated from top 5 universities, compared to 32 percent of Ph.D.s during the 2000s. A similar pattern is observed for the prestige of doctoral institutions. While the number of new Ph.D.s from non-SKY domestic institutions more than doubled over the periods, from 19 percent to 45 percent, the number who received their doctoral degrees from the world’s top 100 universities declined by over half, from 29 percent to 12 percent. In terms of academic discipline, about one quarter of Ph.D.s in Korea are in engineering, with another quarter in the natural sciences. Roughly one in seven doctoral holders is either from the social sciences or humanities.

**Fig 2:**
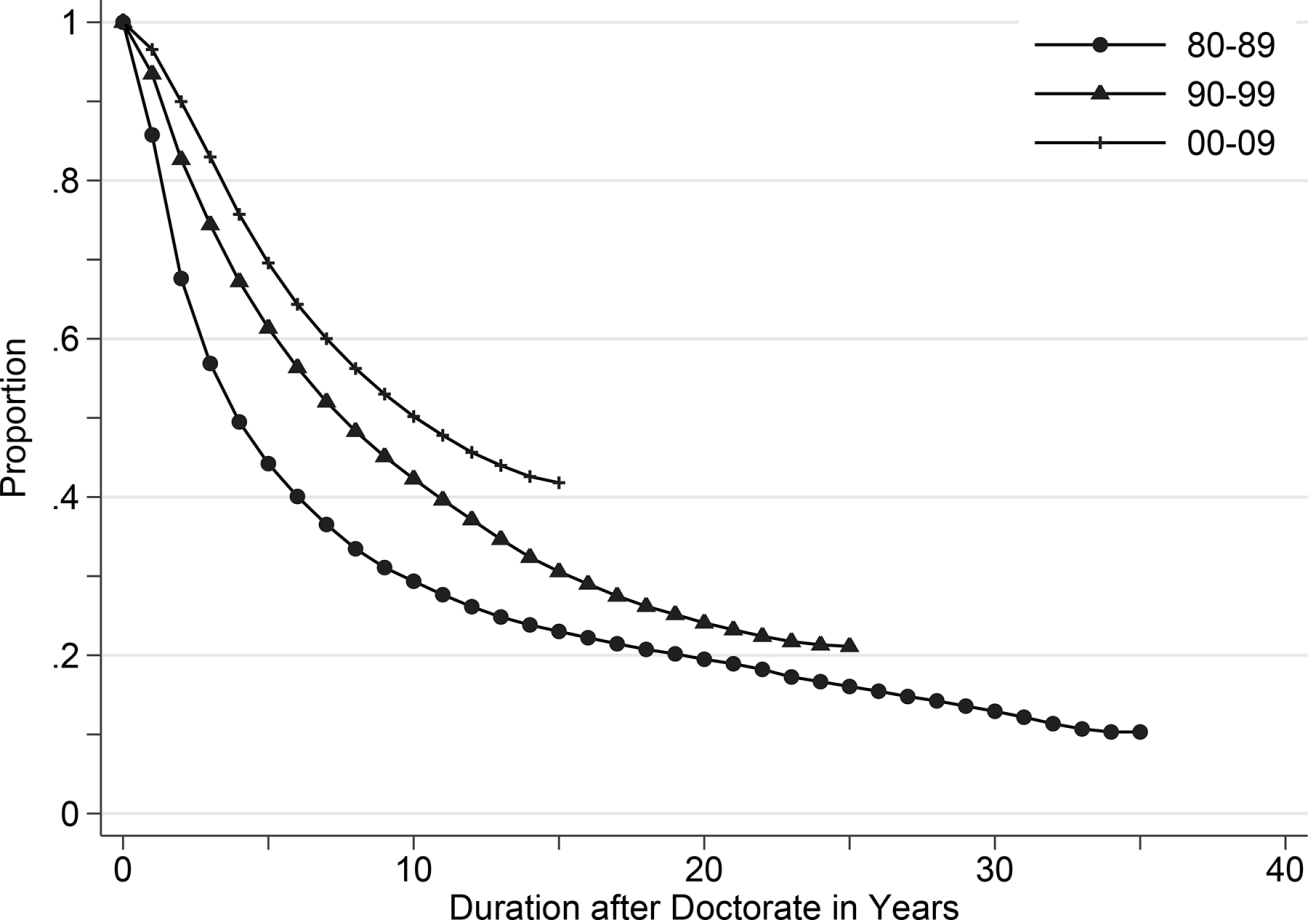
Proportion of Doctoral Degree Holders Not Hired as Tenure-Track Faculty, by Doctoral Cohort.

Change in the risk of academic employment over time is presented in Fig 2, where the yearly proportion of Ph.D.s who did not succeed in getting an academic position is plotted. The figure displays the tightening of the job market that occurred in the 1990s, and the slowing of this trend in the 2000s. The change in employment rates was substantial. For the 1980–1989 cohort, the median survival time (i.e., half of the sample succeeded in getting an academic position) was approximately 5 years, whereas it became almost 9 years and 11 years for the 1990–1999 and 2000–2009 cohorts, respectively.

Table 2 presents the results of the event history multiple logistic regression analysis. For the most part, these effects are in accordance with prior research, and because the coefficients show similar patterns across the three cohorts, we interpret the result by using the 1980–1989 cohort as an example. The results indicate that, net of other factors, the risk of academic appointment for female Ph.D.s is 20.6 percent ((1-exp[-0.231]) × 100) lower than their male peers, confirming the widespread and persistent gender inequality in the academic profession, not only in Korea [21] but also in other countries [28], [38]. Also, each one-year increase in age at doctoral receipt decreases the odds of transitioning to an academic position by 2.2 percent.

**Table 2:** Coefficients from the Logistic Regression Analysis of Timing of First Tenure-Track Academic Position on Selected Independent Variables, by Doctoral Cohorts: Korea Researcher Information (KRI) Database, 1980–2009. Standard errors are in parentheses. For number of publications as a doctoral student, the omitted category is “no publications”; for prestige of undergraduate institution, the omitted category is “below top 30 university”; for prestige of graduate institution, the omitted category is “domestic, non-SKY university.” SKY universities also include KAIST and POSTECH. All models control for academic discipline fixed-effects and current year.

	1980–89		1990–99		2000–09	
	Coef.	S.E.	Coef.	S.E.	Coef.	S.E.
Female	-0.231^***^	(0.053)	-0.557^***^	(0.027)	-0.452^***^	(0.025)
Age at Receipt of Doctorate	-0.023^***^	(0.008)	-0.015^***^	(0.004)	-0.004	(0.004)
Years between Baccalaureate and Doctorate	-0.029^***^	(0.008)	-0.015^***^	(0.005)	-0.013^***^	(0.004)
*Number of Publications as Doctoral Student*						
1 Publication	0.230^***^	(0.057)	0.183^***^	(0.026)	0.230^***^	(0.029)
2 Publications	0.160^**^	(0.074)	0.284^***^	(0.032)	0.301^***^	(0.031)
3+ Publications	0.290^***^	(0.061)	0.324^***^	(0.026)	0.527^***^	(0.023)
*Prestige of Undergraduate Institution*						
Top 30 University	0.255^***^	(0.059)	0.072^***^	(0.027)	0.129^***^	(0.027)
Top 10 University	0.262^***^	(0.062)	0.155^***^	(0.031)	0.447^***^	(0.031)
Top 5 University	0.228^***^	(0.054)	0.226^***^	(0.028)	0.576^***^	(0.028)
*Prestige of Graduate Institution*						
Domestic, SKY University	0.020	(0.054)	0.094^***^	(0.028)	0.155^***^	(0.029)
Abroad, Non-World Top 100 University	0.230^***^	(0.050)	0.344^***^	(0.025)	0.720^***^	(0.029)
Abroad, World Top 100 University	0.234^***^	(0.052)	0.485^***^	(0.027)	1.120^***^	(0.031)
Time	-0.180^***^	(0.013)	0.088^***^	(0.008)	0.266^***^	(0.012)
Time^2^	0.002^***^	(0.000)	-0.004^***^	(0.000)	-0.021^***^	(0.001)
Constant	-0.280	(0.452)	-1.623^***^	(0.237)	-4.025^***^	(0.265)
Number of person-years	61,618		224,231		221,361	
Number of persons	6,577		22,712		27,938	
Log-likelihood	-17,502		-56,658		-47,594	

^***^ p<0.01

^**^ p<0.05.

Consistent with past research showing a negative effect of the time elapsed between baccalaureate and doctoral degree attainment on the likelihood of getting an academic appointment [3], [51], [33], our results suggest that one additional year spent in graduate school reduces the odds of academic employment by 2.9 percent. This may indicate that hiring institutions use time spent in graduate school as a proxy for graduate school performance and, hence, future productivity.

In line with the universalistic principle, our results indicate that having published at least one article in KCI or SCI (SSCI) journals significantly raises the odds of academic appointment. Specifically, the estimated annual odds of getting an academic position are 25.8, 17.3, and 33.7 percent higher for those who published one, two, or three or more articles, respectively, compared with those without any publications. Our results also provide support for the particularism model because increases in the prestige of undergraduate as well as graduate institutions significantly raises the odds of academic appointment. For example, the annual odds of academic appointment for Ph.D.s who obtained bachelor’s degrees from top 5 universities are about 25.7 percent higher than for those who graduated from top 30 or lower universities. Similarly, doctoral degrees from the world’s top 100 universities are associated with a 26.3 percent increase in the odds of academic employment relative to those from domestic, non-SKY universities. The relative importance of predictors was examined by standardizing all independent variables, and the results are presented in Table A in S1 File.

The main focus of the current analysis is to identify how these effects have changed over time; this can be accomplished by comparing coefficients for one variable across the cohorts in Table 2. The gender coefficient suggests that female Ph.D.s who obtained doctoral degrees during the 1990s were 42.7 percent less likely to land an academic position than their male counterparts, indicating that gender discrimination was higher during the 1990s than the 1980s. Perhaps due to the significant increase during the early 2000s in requests for policies aimed at enhancing gender equity in Korea [52], the gender discrepancy had dropped to 36.4 percent during the 2000s. Recent cohorts of female Ph.D.s are still significantly disadvantaged in the academic labor market, albeit to a lesser extent than in the 1990s.

Relative to the 1980s, the effect of age at doctoral receipt decreased in the 1990s and became insignificant during the 2000s. This may be related to the increase in the average age of Ph.D. recipients in more recent decades. Similar to the gender discrepancy patters outlined above, the effect of years spent in graduate school declined over time but remained significant throughout the periods under study. This decline may be due to increasingly standardized doctoral curriculums across institutions, and to a smaller variation in the years to complete the program within an academic discipline [9].

In contrast to these other trends that experienced a decline over the periods analyzed, the effect of number of publications as a Ph.D. student ‒ one of the most important criteria emphasized by the universalism model ‒ consistently increased over time. For instance, while those who published three or more articles were 33.7 percent more likely to get an academic position than those without any publications during the 1980s, similar scholars were 69.3 percent more likely during the 2000s. Similarly, the effect of particularistic factors–specifically, the prestige of undergraduate and graduate institutions–increased over the same period. However, the particularistic factors increases were much larger than those for universalistic factors. For example, while doctoral degrees from the world’s top 100 universities were associated with a 26.3 percent increase in the odds of obtaining an academic position during the 1980s, degree holders from these universities in the 2000s were 3.06 times more likely to be employed as academic faculty compared with those who earned their Ph.D.s in domestic, non-SKY universities. Those who obtained Ph.D.s from non-top 100 world universities were roughly two times more likely to become academic faculty compared with those who earned degrees from domestic, non-SKY universities. Even among domestic institutions, stratification increased during the 1990s. That is, during the 1980s, there were no significant differences between SKY and non-SKY universities on the likelihood of becoming a professor. However, since the 1990s, doctoral degrees from SKY universities have significantly increased the odds of academic employment; in the 2000s, obtaining a Ph.D. from a SKY university was associated with a 16.8 percent increase in the odds of academic employment.

**Fig 3:**
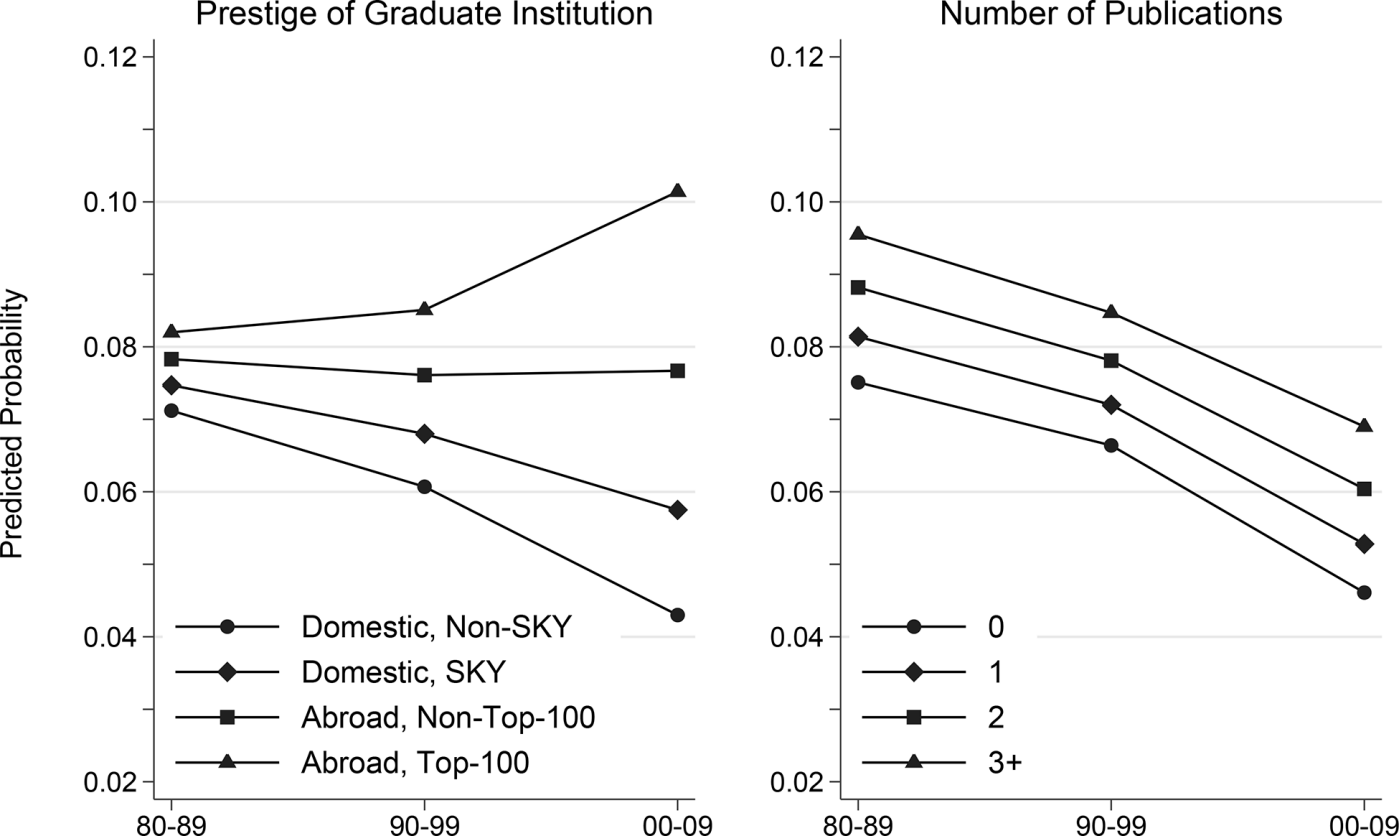
Predicted Probability of Getting a First Tenure-Track Academic Position, by Prestige of Graduate Institution, Number of Publications, and Doctoral Cohort: Korea Researcher Information (KRI) Database, 1980–2009.

Fig 3 illustrates the predicted probabilities of getting a first tenure-track position by prestige of graduate institution, a prominent particularistic factor, and by number of publications, a prominent universalistic factor, delineated by doctoral cohort. This graph is derived from the estimated coefficients in Table 2, with all covariates set to their mean value except the prestige of doctoral institution and number of publications. The graph shows that the probability of getting a tenure-track position increases as the prestige of doctorate-granting institution across all cohorts, with the highest for world top-100 universities and the lowest for domestic non-SKY universities. However, the probability increased for those with doctoral degrees from the world’s top-100 universities over the periods, particularly during the 2000s, while it decreased for those who graduated from domestic non-SKY universities over the same periods. As a result, the gap in the probability of getting an academic position by the prestige of doctorate-granting institution has widened, and the discrepancy was most pronounced during the 2000s. On the other hand, the probability of getting an academic position by the number of publications decreased over the same periods, though a greater number of publications is associated with a higher probability of employment in all periods. Also, the gaps in the probability by number of publications remained more or less the same during the periods. Overall, this pattern clearly demonstrates that inequalities by a particularistic factor grew between 1980s and 2000s whereas discrepancies by a universalistic factor changed to a limited degree during the same periods.

Thus far, we have discussed changes in the determinants of entry into the academic career across all disciplines. However, a number of scholars contend that disciplines differ in their emphasis on universalistic versus particularistic criteria when evaluating candidates. For instance, Hargens and Hagstrom found that disciplines with low level of disciplinary consensus, such as the social sciences, rely more on particularistic criteria than disciplines with high consensus [32]. Whitley [53] also contended that disciplines with standardized procedures for evaluation, such as the natural sciences or engineering, are likely to place greater emphasis on individual performance for employment than fields lacking such standardized procedures [54].

**Fig 4:**
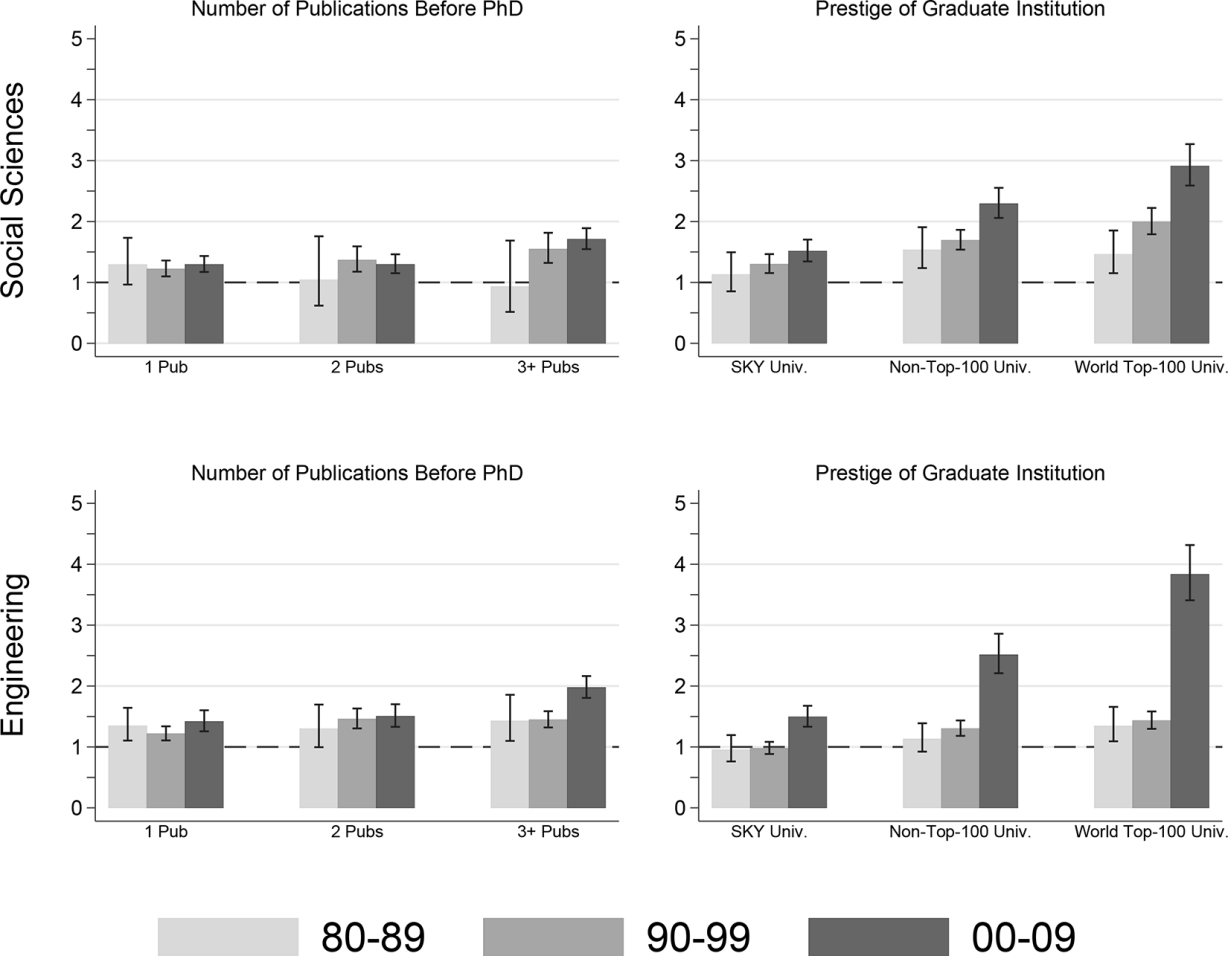
Odds Ratios from the Discrete-Time Event History Regression of Entry into the Academic Career on Selected Independent Variables, by Doctoral Cohorts: Social Sciences and Engineering. Values greater than 1.00 indicate a positive association, while values less than 1.00 indicate a negative association. Error bars are 95% confidence intervals. All models control for gender (female), age at receipt of doctorate, years between baccalaureate and doctorate, prestige of undergraduate institution, academic discipline, year at observation, time, and time squared.

To assess these hypotheses, we re-estimated the models in Table 2 separately for the seven broadly defined academic disciplines; we present the results for social sciences and engineering, to represent the hard sciences and soft sciences, respectively. Odds ratios for the number of publications as a Ph.D. student and prestige of the graduate institution are presented because these two variables presumably are the most representative elements of the universalistic and particularistic models, respectively. The results are illustrated in Fig 4.

Results indicate that hypothesized differences in hiring practices between hard and soft sciences are not found in the Korean context. In both social sciences and engineering, prestige of the doctoral institution has a much larger effect on the odds of academic appointment than does the number of publications before Ph.D. receipt, although the effects of both variables have increased linearly over time. Furthermore, comparing the odds ratios suggests that, in Korea, doctoral degrees from foreign institutions are somewhat more highly regarded in engineering than in the social sciences. For example, among the 2000–2009 cohort, those who obtained social science doctorates from the world’s top 100 universities are about three times more likely to get an academic position than those who graduated from domestic, non-SKY universities; in contrast, those who earned engineering doctorates from the world’s top 100 universities are about four times more likely to get an academic position.

## Discussion and Conclusion

Despite substantial research on the numerous determinants of entry into the academic career, little is known about how these determinants have changed over time or how they vary by academic discipline. This discrete-time event history analysis of transitions to the role of assistant professor for a large sample of Korean scholars who earned doctorates over the past three decades yielded results that are relatively consistent with past studies: both particularistic and universalistic elements can strongly affect subsequent career success. More specifically, in line with [21], the present study finds that female Ph.D.s in Korea are significantly less likely to obtain a tenure-track faculty job than their male counterparts, even after controlling for other factors, including discipline-specific characteristics. Moreover, it appears that the observed gender effect is not attributable to the increasing proportion of doctorates being earned by women over the three decades examined in this study (see Table B in S1 File). In addition, both age at doctoral receipt and the time elapsed between the bachelor’s degree and doctoral degree significantly reduce the probability of academic employment, which is also consistent with past literature [3], [32], [33].

More importantly, the prestige of undergraduate and graduate institutions, as well as the number of publications as a graduate student, is significantly associated with the likelihood of academic employment. That is, degrees from more prestigious undergraduate and graduate institutions, particularly from the SKY universities in Korea or overseas institutions, significantly elevates the odds of academic appointment. At the same time, each additional publication significantly increases the odds of success in the job search. These results imply that both the principles of universalism and particularism operate in academic institutions in Korea.

The current study also showed important historic variations in the impact of several well-established determinants of academic employment. First, our analyses indicate that the effect of the prestige of one’s doctoral institution increased to a much greater extent than did the influence of one’s publication record. This is particularly noteworthy because it implies that the impact of a particularistic element on one of the most crucial academic rewards ‒ employment in higher education ‒ has increased to a larger degree than the effect of any universalistic characteristic.

While the extant literature suggests that the effect of particularistic characteristics should diminish as hiring processes are formalized and the ambiguity of evaluation standards is reduced [26], evidence also suggests that, in Korea, academic hiring processes have become more transparent and evaluation standards have been increasingly regulated over time [19]. This apparent contradiction may be attributable to the historical development of higher education in Korea. Criticizing the excessive Americanization of faculty members and academic structures in Korea, some leading scholars in the 1980s and early 1990s argued for the indigenization of intellectuals by strengthening graduate education in Korea. Although similar arguments continued to appear sporadically, those efforts ultimately failed, largely due to an emphasis on the globalization of universities.

As movements toward a global economy increased in the late 1990s, Korean universities gravitated toward scholars who could conduct globalized, cooperative research; give lectures in English; and publish papers in international journals (which are predominantly English-language). In the early 2000s, the Korean government reinforced this trend by allocating research grants based on university rankings, which, in turn, are based on globalization indices, including the number of SCI (SSCI) publications per faculty member [27]. Therefore, an emphasis on the global competiveness of scholars led to increasing demand for English-speaking faculty, who are predominantly educated in the U.S. In a sense, from the standpoint of mainstream academics in Korea, doctoral degrees earned in foreign countries are equivalent to reliable evidence of academic potential and future productivity, rather than a simple manifestation of preference based on particularism. Consequently, the discrepancy in the likelihood of academic employment between doctorates from globally recognized universities and those from domestic institutions has grown over time.

Furthermore, our analyses by academic discipline indicate that the effect of particularistic characteristics has grown to a larger extent in engineering and the natural sciences than in the social sciences and humanities. This result contradicts prior literature, which argued that academic fields characterized by more highly developed scientific paradigms tend to show stronger associations between universalistic determinants and scholarly rewards than fields with less developed scientific paradigms, due to their greater predictability in evaluation [55], [26], [28].

This unexpected finding may be explained by Korea’s increased intellectual dependence on Western countries, particularly the U.S. In the natural sciences and engineering, demand to learn from Western countries increased, rather than decreased, in more recent decades, largely because these fields began to be regarded as the best means for national economic development as the knowledge economy emerged in the 1990s. Furthermore, research in the natural sciences and engineering often requires substantial funding for equipment and research assistants and is conducted by teams, whereas scholars in the social sciences or humanities tend to work alone and often need only computers and books [56]. Thus, discrepancies in research support between Korea and countries with more advanced scientific communities may be more pronounced in the natural sciences and engineering than in the social sciences and humanities. Indeed, one study of engineering Ph.D.s found that the primary reason students entered graduate schools in overseas institutions was to learn state-of-the-art techniques unavailable in Korea [20]. Another study found that 77.6 percent of professors and academic administrators view the most significant problem of graduate education in Korea as the shortage of facilities and equipment [25]. As in other scientific fields in Korea, the vast majority of professors in engineering and the natural sciences obtained their doctorates from U.S. institutions, and these professors are often influential. To the extent that these elite professors prefer newly minted Ph.D.s from U.S. institutions, the link between particularistic characteristics, especially degrees from globally renowned universities, and employment decisions may continue to tighten over time.

Finally, the present study also lends partial support to [38], who argued that gender differences in science are diminishing. Our results indicate that the female disadvantage was stronger in the 1990s than in the 1980s, and, despite some narrowing of the gap during the 2000s, it never returned to the level observed during the 1980s. Hence, as [21] found, female Ph.D.s are still substantially disadvantaged relative to their male peers in the academic job market in Korea, though this has improved somewhat in recent decades. Kim and Shin argued that, perhaps, decreasing gender discrimination in the Korean academic market is due to the implementation of employment quotas for female professors at national universities during the early 2000s [17].

As with any research study, the current study is not without limitations. First, due to data limitations, the present study could not examine the effect of graduate advisors or mentors on the likelihood of academic appointment. A number of studies have indicated that the prestige or productivity of one’s graduate advisors, especially the chairperson of one’s dissertation committee, is significantly associated with the probability of landing a tenure-track academic position. This effect may operate through introducing the doctoral student to other influential professors or because the eminence of the advisor may be interpreted as a proxy for the productivity of the applicant [57], [14]. Nonetheless, several studies noted that the effect of graduate department prestige outweighs mentor eminence [3], [31], and, thus, omission of information about the advisor’s eminence would not have changed our overall findings.

Second, the current analyses could not investigate the effect of postgraduate training on the odds of academic employment, due to lack of such information in the KRI. Past studies found that postgraduate training can affect the likelihood of academic employment [13]. Postgraduate positions may weaken individuals’ ties with their doctoral institution and lead to the development of new ties at the postgraduate institution. Hence, young scholars from less prestigious doctoral institutions may be able to increase their desirability through postgraduate training. Also, by securing additional time for publications, students working as postdoctoral fellows may have more time to establish themselves as researchers.

Third, the present study could not examine the effect of publication quality, which is typically measured by the number of times an article is cited. Although prior research has found a significant influence of the number of citations on academic labor market outcomes [13], we believe that the quantity of publications alone can effectively represent the effect of scholarly performance on academic appointment, particularly for recent Ph.D.s, given their short time to establish themselves as researchers and be recognized in academia. Nonetheless, publication quality would be an important avenue for future research to examine as a determinant of academic employment.

In an era of globalization, higher education institutions in Korea face unprecedented pressure to improve the quality of education and to recruit and retain the best possible scholars not only from domestic institutions, but also from those in other countries. Thus, an increasing effect of particularistic characteristics on the likelihood of academic appointment would not only weaken the competitiveness of Korean universities in globalized markets over the long run, but also undermine the integrity of the scientific community as a whole. Ultimately, if these patterns persist, higher education for future generations might be at stake. Therefore, universities and their constituents should reflect on the fundamental principles of the institution of science, and collective efforts should be made to restore the spirit of universalism to academic hiring decisions.

## Supporting Information

S1 File Supporting Information and Tables. Unstandardized and Standardized Coefficients from the Logistic Regression Analysis of Timing of First Tenure-Track Academic Position on Selected Independent Variables, by Doctoral Cohorts: Korea Researcher Information (KRI) Database, 1980–2009 **(Table A)**. Coefficients from the Logistic Regression Analysis of Timing of First Tenure-Track Academic Position on Selected Independent Variables Including the Percent of Female Doctors Within Academic Discipline, by Doctoral Cohorts: Korea Researcher Information (KRI) Database, 1980–2009 **(Table B)**. Estimates of Model Fit for Models Excluding Age at Receipt of Doctorate and Years Between Baccalaureate and Doctorate by Doctoral Cohort **(Table C)**.
(DOCX)

S2 File Data Set Used in the Analysis. (ZIP)
