# Supplementary material for: Trends in Determinants of Entry into the Academic Career: The Case of South Korea, 1980-2010
Source: PLoS One. 2015 Oct 28;10(10):e0141428. doi: 10.1371/journal.pone.0141428 (PMC4625013; doi:10.1371/journal.pone.0141428)
Supplement: S1 File — Unstandardized and Standardized Coefficients from the Logistic Regression Analysis of Timing of First Tenure-Track Academic Position on Selected Independent Variables, by Doctoral Cohorts: Korea Researcher Information (KRI) Database, 1980–2009 (Table A). Coefficients from the Logistic Regression Analysis of Timing of First Tenure-Track Academic Position on Selected Independent Variables Including the Percent of Female Doctors Within Academic Discipline, by Doctoral Cohorts: Korea Researcher Information (KRI) Database, 1980–2009 (Table B). Estimates of Model Fit for Models Excluding Age at Receipt of Doctorate and Years Between Baccalaureate and Doctorate by Doctoral Cohort (Table C). (DOCX) [file pone.0141428.s001.docx]

**Supporting Information for:**

**Trends in Determinants of Entry into the Academic Career: The Case of South Korea, 1980-2010**

Keuntae Kim and Jong-Kil Kim

*The Most Important Factors for Faculty Appointment*

One might wonder which factor has the most significant impact on the likelihood of faculty hiring. In order to compare the effects of variables measured in different metrics, we standardized each covariate to have a mean of 0 and a standard deviation of 1 [1], [2].

Table A presents the standardized as well as unstandardized coefficients from the logistic regression models corresponding to the results presented in Table 2 of the main text. Results indicated that, in addition to time-to-degree, holding a doctoral degree from a world top-100 university is one of the most important predictors of the likelihood of academic employment. This pattern held for all cohorts, and the main text shows that the effect size increases across doctoral cohorts. The second most influential factor appears to be the prestige of one’s undergraduate institution. In particular, among the two most recent doctoral cohorts, graduating from a top-5 Korean university yields a stronger influence on academic employment than having more than three publications. In addition, it appears that the gender effect in the Korean academic job market is stronger than the effect of number of publications. Overall, the results for standardized coefficients are similar to findings from the main analyses using unstandardized coefficients.

*Gender Effects*

It is possible that the observed gender effect on the odds of academic employment reflects the fact that more females than males are obtaining doctoral degrees, rather than real structural changes in the academic job market. To account for the increasing number of female doctorates over time, we constructed a measure that captures the percentage of doctoral recipients who are female in a given academic field and year. We re-estimated each model including this variable to examine the gender effect net of the proportion of new doctoral degree earners who are female in a given field. The results, presented in Table B, reveal that the gender coefficients remained virtually unchanged for all cohorts, even when controlling for percentage female doctorates. This implies that the observed gender effect shown in the main analysis is not attributable to the increasing number of female doctorates over the three decades examined in this study.

*The Impact of Time to PhD and Age upon PhD Conferral*

It is possible that time to PhD and age upon PhD conferral are weak proxy measures for academic productivity and should be removed from the model. To determine whether these variables should be excluded, we followed an established model selection technique, comparing the model fits across four alternative model specifications: (1) the full model (results reported in Table 2 of the main text); (2) a restricted model excluding age at receipt of doctorate; (3) a restricted model excluding years between baccalaureate and doctorate; and (4) a restricted model excluding both variables. If we observe that model fit significantly worsens as compared to the full model (i.e., full model) when we exclude the time-to-degree or age-upon-conferral variables, we will interpret this as evidence that those variables should be included in the model. We relied on two information criteria (IC) to assess comparative model fit: Bayesian information criterion (BIC) and Akaike information criterion (AIC) [3]. Among a set of models, the model with the lowest AIC or BIC is preferred. AIC and BIC are calculated as follows:

AIC = -2*ln(likelihood) + 2*k

BIC = -2*ln(likelihood) + ln(N)*k

where *k* denotes the number of estimated parameters and *N* refers to the number of observations. Furthermore, we use model selection guidelines suggested by Raftery: a decline of 0-2 IC is weak evidence of fit improvement, a decline of 2-6 IC is positive evidence, a decline of 6-10 IC is strong evidence, and a decline of greater than 10 IC is very strong evidence for model fit improvement [3].

Results (see Table C) indicated that, when we excluded age at PhD conferral from the model estimated on the 1980-89 cohort data, the AIC and BIC respectively increased by 386.25 and 388.81. Because these values are far greater than 10, we concluded that model fit substantially deteriorates when excluding this variable, as compared to the full model. When we excluded years to doctoral degree, the AIC and BIC values increased, again indicating that the model fit significantly worsens when excluding this variable from the model. Not surprisingly, model fit was worst when we dropped both variables from the model. The same patterns were observed for the other two doctoral cohorts. Because the weight of evidence suggests that including both variables significantly improves the explanatory power of the model estimating the determinants of academic employment among PhDs in Korea.

**References**

1. Kaufman RL. Comparing Effects in Dichotomous Logistic Regression: A Variety of Standardized Coefficients. Soc Sci Q. 1996 Mar 1;77(1):90–109.

2. Menard S. Six Approaches to Calculating Standardized Logistic Regression Coefficients. Am Stat. 2004 Aug 1;58(3):218–23.

3. Raftery AE. Bayesian Model Selection in Social Research. Sociol Methodol. 1995 Jan 1;25:111–63.

| **Table A. Unstandardized and Standardized Coefficients from the Logistic Regression Analysis of Timing of First Tenure-Track Academic Position on Selected Independent Variables, by Doctoral Cohorts: Korea Researcher Information (KRI) Database, 1980-2009** | | | | | | | | |
| --- | --- | --- | --- | --- | --- | --- | --- | --- |
|  | 1980-89 | |  | 1990-99 | |  | 2000-09 | |
|  | *b* | *B* |  | *b* | *B* |  | *b* | *B* |
| Female | -0.231*** | -0.077 |  | -0.557*** | -0.229 |  | -0.452*** | -0.209 |
| Age at Receipt of Doctorate | -0.023*** | -0.108 |  | -0.015*** | -0.066 |  | -0.004 | -0.021 |
| Years between Baccalaureate and Doctorate | -0.029*** | -0.128 |  | -0.015*** | -0.058 |  | -0.013*** | -0.059 |
| *Number of Publications as Doctoral Student* |  |  |  |  |  |  |  |  |
| 1 Publication | 0.230*** | 0.053 |  | 0.183*** | 0.058 |  | 0.230*** | 0.079 |
| 2 Publications | 0.160** | 0.028 |  | 0.284*** | 0.070 |  | 0.301*** | 0.097 |
| 3+ Publications | 0.290*** | 0.063 |  | 0.324*** | 0.103 |  | 0.527*** | 0.248 |
| *Prestige of Undergraduate Institution* |  |  |  |  |  |  |  |  |
| Top 30 University | 0.255*** | 0.099 |  | 0.072*** | 0.032 |  | 0.129*** | 0.057 |
| Top 10 University | 0.262*** | 0.093 |  | 0.155*** | 0.056 |  | 0.447*** | 0.150 |
| Top 5 University | 0.228*** | 0.114 |  | 0.226*** | 0.110 |  | 0.576*** | 0.261 |
| *Prestige of Graduate Institution* |  |  |  |  |  |  |  |  |
| Domestic, SKY University | 0.020 | 0.008 |  | 0.094*** | 0.041 |  | 0.155*** | 0.069 |
| Abroad, Non-World Top 100 University | 0.230*** | 0.103 |  | 0.344*** | 0.146 |  | 0.720*** | 0.265 |
| Abroad, World Top 100 University | 0.234*** | 0.103 |  | 0.485*** | 0.179 |  | 1.120*** | 0.326 |
| Time | -0.180*** | -1.514 |  | 0.088*** | 0.492 |  | 0.266*** | 0.927 |
| Time^2^ | 0.002*** | 0.498 |  | -0.004*** | -0.463 |  | -0.021*** | -1.009 |
| Constant | -0.280 |  |  | -1.623*** |  |  | -4.025*** |  |
| Number of person-years | 61,618 | |  | 224,231 | |  | 221,361 | |
| Number of persons | 6,577 | |  | 22,712 | |  | 27,938 | |
| Log-likelihood | -17,502 | |  | -56,658 | |  | -47,594 | |
| *Note*: *b* refers to unstandardized coefficients and *B* denotes standardized coefficients. For number of publications as a doctoral student, the omitted category is “no publications”; for prestige of undergraduate institution, the omitted category is “below top 30 university”; for prestige of graduate institution, the omitted category is “domestic, non-SKY university.” SKY universities also include KAIST and POSTECH. All models control for academic discipline fixed-effects and current year. | | | | | | | | |
| *** *p*<0.01, ** *p*<0.05, * *p*<0.1 | | | | | | | | |

| **Table B. Coefficients from the Logistic Regression Analysis of Timing of First Tenure-Track Academic Position on Selected Independent Variables Including the Percent of Female Doctors Within Academic Discipline, by Doctoral Cohorts: Korea Researcher Information (KRI) Database, 1980-2009** | | | | | | | | |
| --- | --- | --- | --- | --- | --- | --- | --- | --- |
|  | 1980-89 | |  | 1990-99 | |  | 2000-09 | |
|  | Coef. | S.E. |  | Coef. | S.E. |  | Coef. | S.E. |
| Female | -0.229*** | (0.053) |  | -0.560*** | (0.027) |  | -0.450*** | (0.025) |
| Percent of Female | -0.094 | (0.312) |  | 0.481* | (0.247) |  | -1.381*** | (0.458) |
| Age at Receipt of Doctorate | -0.023*** | (0.008) |  | -0.015*** | (0.004) |  | -0.004 | (0.004) |
| Years between Baccalaureate and Doctorate | -0.029*** | (0.008) |  | -0.015*** | (0.005) |  | -0.013*** | (0.004) |
| *Number of Publications as Doctoral Student* |  |  |  |  |  |  |  |  |
| 1 Publication | 0.230*** | (0.057) |  | 0.183*** | (0.026) |  | 0.230*** | (0.029) |
| 2 Publications | 0.160** | (0.074) |  | 0.284*** | (0.032) |  | 0.303*** | (0.031) |
| 3+ Publications | 0.290*** | (0.061) |  | 0.323*** | (0.026) |  | 0.527*** | (0.023) |
| *Prestige of Undergraduate Institution* |  |  |  |  |  |  |  |  |
| Top 30 University | 0.255*** | (0.059) |  | 0.073*** | (0.027) |  | 0.129*** | (0.027) |
| Top 10 University | 0.263*** | (0.062) |  | 0.156*** | (0.031) |  | 0.446*** | (0.031) |
| Top 5 University | 0.229*** | (0.054) |  | 0.226*** | (0.028) |  | 0.576*** | (0.028) |
| *Prestige of Graduate Institution* |  |  |  |  |  |  |  |  |
| Domestic, SKY University | 0.020 | (0.054) |  | 0.095*** | (0.028) |  | 0.155*** | (0.029) |
| Abroad, Non-World Top 100 University | 0.230*** | (0.050) |  | 0.344*** | (0.025) |  | 0.719*** | (0.029) |
| Abroad, World Top 100 University | 0.233*** | (0.052) |  | 0.484*** | (0.027) |  | 1.121*** | (0.031) |
| Time | -0.180*** | (0.013) |  | 0.088*** | (0.008) |  | 0.266*** | (0.012) |
| Time^2^ | 0.002*** | (0.000) |  | -0.004*** | (0.000) |  | -0.021*** | (0.001) |
| Constant | -0.209 | (0.511) |  | -2.038*** | (0.319) |  | -2.790*** | (0.487) |
| Number of person-years | 61,618 | |  | 224,231 | |  | 221,361 | |
| Number of persons | 6,577 | |  | 22,712 | |  | 27,938 | |
| Log-likelihood | -17,502 | |  | -56,656 | |  | -47,589 | |
| *Note*: Standard errors in parentheses. For number of publications as a doctoral student, the omitted category is “no publications”; for prestige of undergraduate institution, the omitted category is “below top 30 university”; for prestige of graduate institution, the omitted category is “domestic, non-SKY university.” SKY universities also include KAIST and POSTECH. All models control for academic discipline fixed-effects and current year. | | | | | | | | |
| *** *p*<0.01, ** *p*<0.05, * *p*<0.1 | | | | | | | | |

| **Table C. Estimates of Model Fit for Models Excluding Age at Receipt of Doctorate and Years Between Baccalaureate and Doctorate by Doctoral Cohort** | | | | | | |
| --- | --- | --- | --- | --- | --- | --- |
| Doctoral Cohort | Model |  | AIC | AIC Difference | BIC | BIC Difference |
| 1980-89 | A: Full model |  | 35311.50 | --- | 36701.92 | --- |
|  | B: A - Age at Receipt of Doctorate |  | 35697.75 | 386.25 | 37090.73 | 388.81 |
|  | C: A - Years between Baccalaureate and Doctorate |  | 36023.08 | 711.58 | 37407.91 | 705.99 |
|  | D: A - (Age at Receipt of Doctorate & Years between Baccalaureate and Doctorate) |  | 36571.69 | 1260.19 | 37959.60 | 1257.68 |
|  |  |  |  |  |  |  |
| 1990-99 | A: Full model |  | 113622.00 | --- | 115201.00 | --- |
|  | B: A - Age at Receipt of Doctorate |  | 115708.90 | 2086.90 | 117280.00 | 2079.00 |
|  | C: A - Years between Baccalaureate and Doctorate |  | 115299.00 | 1677.00 | 116869.90 | 1668.90 |
|  | D: A - (Age at Receipt of Doctorate & Years between Baccalaureate and Doctorate) |  | 117553.00 | 3931.00 | 119116.00 | 3915.00 |
|  |  |  |  |  |  |  |
| 2000-09 | A: Full model |  | 95470.01 | --- | 96923.37 | --- |
|  | B: A - Age at Receipt of Doctorate |  | 97559.94 | 2089.93 | 99004.82 | 2081.45 |
|  | C: A - Years between Baccalaureate and Doctorate |  | 95970.08 | 500.07 | 97414.02 | 490.65 |
|  | D: A - (Age at Receipt of Doctorate & Years between Baccalaureate and Doctorate) |  | 98110.65 | 2640.64 | 99546.08 | 2622.71 |
| *Note*: Full model refers to Table 2 in the main text. | | | | | | |
